# Disseminated TB With IRIS Presenting as a Pancreatic Mass in Newly Diagnosed HIV: A Case Report

**DOI:** 10.1093/ofid/ofae746

**Published:** 2024-12-21

**Authors:** Nina Akbar, Peter Mariuz

**Affiliations:** Division of Infectious Disease, Nuvance Health/Norwalk Hospital, Norwalk, Connecticut, USA; Division of Infectious Disease, University of Rochester Medical Center, Rochester, New York, USA

**Keywords:** geriatric, HIV: co-infection, host immunity, IRIS, pancreatic TB

## Abstract

Pancreatic tuberculosis (TB) is an uncommon extrapulmonary presentation of TB. Identification of coinfection with HIV may unmask not only disseminated TB but also immune reconstitution inflammatory syndrome (IRIS). We present the case of a 70-year-old Indian woman newly diagnosed with AIDS and pancreatic tuberculosis with miliary disseminated disease. Her clinical course was complicated by IRIS related to HIV-TB coinfection despite sequential and targeted anti-infective therapies. We review the presentation, pathophysiology, and risk factors for developing IRIS with HIV and pancreatic TB.

## CASE REPORT

**Figure 1.**
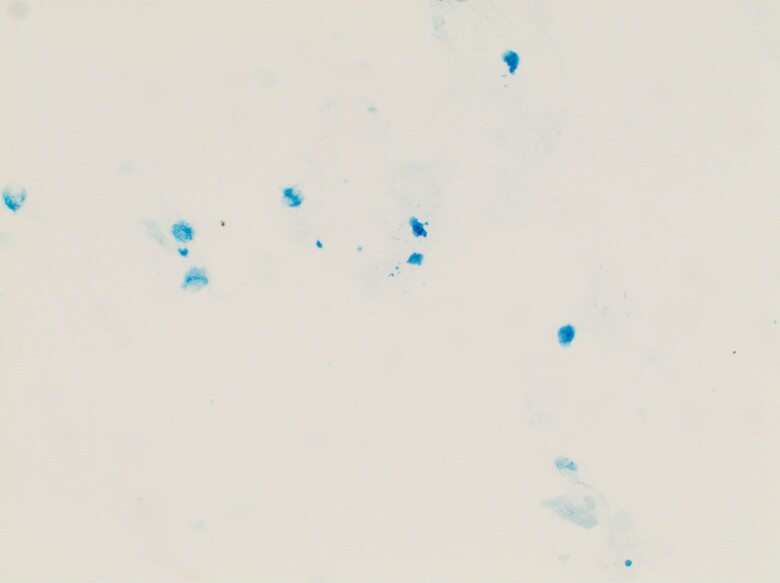
Acid-fast bacillus stain of pancreatic mass biopsy. Courtesy of Dr Bhavna Khandpur, Vice Chairman of Pathology, Nuvance Health/Norwalk Hospital.

A 70-year-old woman with hypothyroidism, who was a housewife from India and would travel to stay in the United States 6 months out of the year, was referred to the infectious disease specialty clinic from her primary care provider and gastroenterologist with unintentional weight loss, anorexia, intermittent nausea/vomiting, headache, myalgia, and generalized weakness. A computed tomography (CT) scan of the abdomen and pelvis with contrast showed a 24 mm × 23 mm × 19 mm ill-defined mass inseparable from the pancreatic neck and medial wall of the descending duodenum with periportal, portacaval, and retroperitoneal adenopathy. Endoscopic ultrasound (EUS) characterized the pancreatic mass as mixed solid/cystic with a fistula draining purulent material into the first part of the duodenum. Biopsy of the pancreatic mass yielded necrotic inflammatory debris with small cellular foci and granulomas but no malignant cells. Acid-fast bacilli (AFB) stain was positive (Figure 1), and culture grew *Mycobacterium tuberculosis* (MTB) complex, susceptible to rifampin, isoniazid, pyrazinamide, and ethambutol. MTB deoxyribonucleic acid (DNA) probe was also positive. QuantiFERON-TB testing was negative. A tuberculin skin test yielded a 5-mm darkened purple macule with central elevation on the left arm. An HIV fourth-generation antigen/antibody screen was repeatedly reactive. CD4 count was 25 cells/μL (3.2%). HIV-1 plasma RNA was 1 100 591 copies/mL (log 6.04).

Three weeks from initial imaging, a follow-up CT scan of the chest, abdomen, and pelvis with contrast showed findings consistent with disseminated TB: heterogeneous mosaic ground-glass lung attenuation with multiple mildly enlarged and shotty axillary lymph nodes, interval decrease in the pancreatic lesion to 9 mm, several pancreatic neck, body, and peripancreatic soft tissue nodules, and retroperitoneal adenopathy with central hypoattenuation. The patient was admitted for 2 weeks to address failure to thrive, during which TB treatment with rifabutin, isoniazid, pyrazinamide, ethambutol, and pyridoxine was initiated.

She returned to the hospital 1 week later with anorexia, generalized weakness, and nausea without abdominal pain. CD4 count was 134 cells/μL (13.3%) with an HIV-1 plasma RNA of 14 126 copies/mL (log 4.15). Bictegravir/emtricitabine/tenofovir alafenamide was started 2 weeks after TB treatment due to risk for IRIS. Six days later, she developed fever to 39.4°C. CT scan of the chest, abdomen, and pelvis with contrast showed multiple punctate pulmonary nodules with upper lobe predominance and slight improvement of bilateral axillary lymphadenopathy, but worsening of retroperitoneal, upper abdominal, and mesenteric lymphadenopathy. There was hyperenhancement and 9.9-mm dilation of the common bile duct (CBD) with focal narrowing above the pancreatic head. Aspartate aminotransferase (AST) was 265 U/L, alanine aminotransferase (ALT) was 168 U/L, alkaline phosphatase was 98 U/L, and total bilirubin was 0.2 mg/dL. Epstein-Barr virus (EBV) reactivated (EBV nuclear antigen, immunoglobulin [Ig] M and IgG titer positive) and cytomegalovirus (CMV) viremia (13 800 copies/mL) was detected. Treatment with oral valganciclovir 900 mg every 12 hours was initiated and later discontinued after 2 weeks as pancytopenia developed. After 3 weeks of combination antiretroviral therapy (cART), CD4 count was 82 cells/μL (16%) with HIV-1 plasma RNA 199 copies/mL (log 2.30). White blood cell count (WBC) was 2.1 × 10^9^/L, hemoglobin was 10.0 g/dL, mean corpuscular volume was 93.8 fL, platelets were 34 × 10^9^/L. Plasma CMV DNA by polymerase chain reaction (PCR) was <200 IU/mL. EBV plasma DNA by PCR was 853 copies/mL (log 2.93). Pyrazinamide and ethambutol were discontinued after 2 months of TB therapy. She was subsequently admitted to the hospital about every 1 to 2 months for anorexia, constipation, and postprandial epigastric and lower abdominal discomfort.

After 5 months of TB treatment and 4 months of cART, her regimen was changed to dolutegravir 50 mg twice a day with emtricitabine-tenofovir disoproxil fumarate 200–300 mg daily because of the potential interaction between bictegravir and rifabutin. After 2 months, CD4 counts improved to 245 copies/mL (9.6%) and her HIV-1 viral load remained suppressed.

At 7.5 months of TB treatment and 6.5 months of cART, CT imaging of the abdomen and pelvis revealed slight enlargement of the pancreatic mass up to 3 cm in diameter, worsening necrotic retroperitoneal lymphadenopathy, and diffuse gastric mural thickening. EUS showed a complex, predominantly cystic but also solid 34 mm × 31 mm irregular mass with internal debris in the pancreatic body. Fine-needle tissue biopsy had mainly granulocyte inflammatory cells, T cells with reversal in the CD4:CD8 ratio, and low cell viability. There was benign acinar tissue, fibrosis, lymphohistiocytic aggregate, and necroinflammatory infiltrate without malignant cells. Tissue staining was positive for a few AFB but negative on Grocott methenamine silver stain and CMV immunostain. Bacterial and AFB cultures were negative. Fungal culture isolated *Candida parapsilosis*, which was not considered clinically relevant, and therapy was deferred.

At 11.5 months of TB treatment and 10.5 months of cART, CD4 count was 148 cells/μL (20.8%) and HIV-1 plasma RNA was 22 copies/μL (log 1.34). The patient had worsening yellow, nonbloody emesis and periumbilical abdominal discomfort with decreased oral intake, for which she was admitted to the hospital. AST was 96 U/L, ALT was 9 U/L, alkaline phosphatase was 83 U/L, total bilirubin was 2.3 mg/dL, and direct bilirubin was 1.7 mg/dL. Magnetic resonance cholangiopancreatography (MRCP) demonstrated narrowing of the CBD within the region of the pancreatic head without evidence of a filling defect. There was a T2 hypointense lesion posterior to the pancreaticoduodenal groove suggestive of granulomatous tissue. There was an interval decrease of collections previously seen in the subpancreatic (5.2 cm to 4.5 cm) and left psoas (3.2 cm to 2.7 cm) regions. The latter was aspirated, and bacterial, fungal, and AFB cultures were negative. There was an interval decrease of mesenteric lymphadenopathy. An endoscopic retrograde cholangiopancreatography (ERCP) showed edema of the major papilla, a single mild biliary stricture in the lower third of the main bile duct, and mild dilation of up to 8 mm of the upper third of the main bile duct. Total bilirubinemia reached to 7.2 mg/dL, and direct bilirubin was 5.4 mg/dL, which subsequently decreased after a 3-mm biliary sphincterotomy was performed with deployment of a 10 mm × 6 cm covered metal biliary stent into the CBD.

CT scan of the chest without contrast revealed an increased, innumerable amount and prominence of nodules throughout the lung parenchyma, moderate-sized left greater than right pleural effusions, upper abdominal ascites, and compressive atelectasis. There was no significant lymphadenopathy. Serial collection of lower respiratory and 2 gastric aspirate sample AFB smears were negative. After consultation with the state health department, TB therapy was broadened for possible multidrug-resistant disseminated TB with levofloxacin, amikacin, amoxicillin-clavulanate, rifampin, and meropenem. Isoniazid was unavailable due to a national shortage. Dobbhoff tube placement facilitated medication and enteric nutrition administration. Normocytic anemia and thrombocytopenia persisted, but leukopenia improved from a nadir of WBC 1.8 × 10^9^/L to a peak of 6.6 × 10^9^/L.

The patient experienced diarrhea and abdominal distention. CT scan of the abdomen and pelvis revealed extensive abdominal peritoneal ascites, haziness of the ascending and proximal transverse colon, circumferential thickening of fluid-filled loops of small bowel in the right lower quadrant, and persistence of the heterogeneous mass along the undersurface of the pancreatic body, which was decreased in size from previous imaging. A diagnostic and therapeutic paracentesis yielded 50 mL of cloudy, turbid yellow fluid with WBC 54/mm^3^ (PMN 7%, lymphocytes 42%, monocytes 49%, eosinophils 1%), RBC <3000/mm^3^, albumin <1.0 g/dL, amylase 37 U/L, glucose 103 mg/dL, LDH 52 U/L, protein 1.6 g/dL, triglycerides 164.0 mg/dL. Final culture results from 1 of the 2 gastric aspirates were positive for *Mycobacterium chelonae*, which was likely an environmental contaminant. The rest of the cultures from the left psoas aspirate, sputum, and ascites fluid were negative.

**Figure 2.**
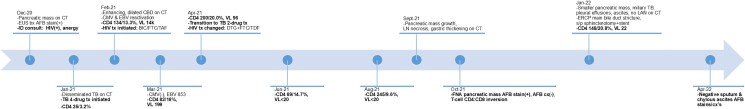
Timeline of clinical course. Abbreviations: AFB, acid-fast bacilli; BIC, bictegravir; CBD, common biliary duct; CMV, cytomegalovirus; CT, computed tomography; cx, culture; DTG, dolutegravir; EBV, Epstein-Barr virus; ERCP, endoscopic retrograde cholangiopancreatography; EUS, endoscopic ultrasound; FNA, fine-needle aspirate; FTC, emtricitabine; ID, infectious disease; LAN, lymphadenopathy; LN, lymph node; s/p, status post; TAF, tenofovir alafenamide; TB, tuberculosis; TDF, tenofovir disoproxil fumarate; tx, therapy; VL, HIV viral load.

The patient became delirious, and her family expressed a preference to focus on quality of life, transitioning to palliative/hospice care. Figure 2 provides a timeline of the clinical course.

## DISCUSSION

Tuberculosis is a common opportunistic infection and leading cause of death in persons with HIV-1 worldwide [1]. Awareness of pancreatic TB may be low, and increasing numbers of immunocompromised patients are presenting with extrapulmonary TB manifestations [2, 3]. Pancreatic tuberculosis mimics malignancy, pseudocyst, and retroperitoneal masses on imaging, necessitating histological or microbiological confirmation as seen in this patient by the presence of granulomas, AFB-positive staining with MTB complex growth in culture, and positive MTB DNA probe from EUS fine-needle biopsy [4–6].

Identification of coinfection with HIV is important as the clinical and diagnostic course may be affected by the degree of immunosuppression and IRIS. T cells are anergic, decreasing the sensitivity of purified protein derivative (≥5 mm denotes a positive result) or QuantiFERON-TB assays [7–9]. This patient presented with a negative QuantiFERON-TB result in the setting of advanced AIDS with an initial low CD4 count of 25 cells/μL (3.2%) and impaired CD4 cell function, reflected by inversion of the CD4:CD8 ratio on fine-needle tissue biopsy of the pancreatic mass despite a CD4 count >200 cells/μL and viral load suppression [10, 11]. An inverted ratio may also indicate immune senescence in an elderly patient and chronic HIV disease [12–14]. Immunosuppression increases the risk for reactivation of latent TB and disseminated disease both in the lungs and extrapulmonary sites. The level of immunosuppression is also influenced by associated opportunistic infections, increasing oxidative stress, chronic inflammation, reduced phagocytic capacity of neutrophils and macrophages, and impaired complement function [1, 12, 13]. As observed by this case, virologic suppression with cART is associated with variable degrees of immune recovery; however, a low CD4 count remains a significant risk factor for TB morbidity and mortality [15]. Immunopathology is decreased in patients with AIDS and TB until chemotherapeutics for both infections augment immune response, giving rise to IRIS [11, 15, 16].

There are 2 forms of TB-IRIS occurring within 3 months of cART initiation: paradoxical—worsening symptoms involving ≥2 disease locations; unmasking—marked intensity of clinical manifestations, especially inflammatory symptoms. TB-IRIS presents with nonspecific systemic illness and lymphadenopathy alongside mimicry by overlapping drug reactions, treatment failure, opportunistic infections, or malignancies. IRIS is the result of a vigorous immune recovery directed toward TB antigens, with disseminated TB being the greatest risk for development of paradoxical TB-IRIS. Often, TB-IRIS is self-limited, though there may be varying mortality reported due to the inconsistencies of defining criteria. There is little evidence on TB-IRIS management. Corticosteroids are noted as first-line therapy from a randomized placebo-controlled trial in South Africa. Nonsteroidal anti-inflammatory drugs may treat symptoms in milder or localized TB-IRIS, yet formal evidence from clinical trials is currently unavailable. There are case reports on the use of other agents like TNF-α inhibitors, thalidomide, montelukast, pentoxifylline, and VEGF inhibitors [17]. The World Health Organization HIV guidelines recommend starting cART within 2 weeks of TB treatment due to the benefits of rapid cART initiation [18].

In this patient, paradoxical TB-IRIS presented during the first 4 weeks of cART with high fever, tachycardia, and weight loss. Worsening of TB features was seen with hyperenhancement and dilation of the CBD with focal narrowing above the pancreatic head, suggestive of resurgence of pancreatic TB manifestations involving the mass and retroperitoneal, abdominal, and mesenteric lymphadenopathy together with continued anorexia, constipation, and epigastric and lower abdominal discomfort. Risk factors for this patient included low CD4 count, disseminated TB, and rapid increase of CD4 counts post-cART [17].

Inability to sustain CD4 counts above 200 cells/μL and TB-IRIS manifesting with abdominal distention from peritoneal ascites, innumerable lung nodules, and bilateral pleural effusions, yet with improvement in the size of the pancreatic mass and lymphadenopathy in the setting of negative respiratory, gastric, and ascitic microbiologic results for TB, argue against treatment failure and development of multidrug resistance [11, 19].

Thus, our case illustrates that patients with pancreatic TB may be presenting as part of a larger, complicated picture with disseminated TB and HIV coinfection, AIDS-related conditions, opportunistic infections, poor nutritional status, advanced age, and impaired host immunity. The course of natural disease, diagnostic studies, and time to clinical response on appropriate HIV and TB treatments may be offset not only by host characteristics but also by IRIS in response to anti-infective therapy and host immunocompetency. There should be a high index of suspicion for HIV-TB coinfection and IRIS in patients from endemic regions with the rare presentation of pancreatic TB.
