# Correction to: Disseminated TB With IRIS Presenting as a Pancreatic Mass in Newly Diagnosed HIV: A Case Report

**DOI:** 10.1093/ofid/ofaf064

**Published:** 2025-02-03

In the original publication of this article [Nina Akbar and Peter Mariuz, Disseminated TB With IRIS Presenting as a Pancreatic Mass in Newly Diagnosed HIV: A Case Report. *Open Forum Infectious Diseases,* Volume 12, Issue 1, January 2025, https://doi.org/10.1093/ofid/ofae746], an error in production incorrectly attributed Figure 2 to: Dr Bhavna Khandpur, Vice Chairman of Pathology, Nuvance Health/Norwalk Hospital. This has been corrected in the version currently available online, and the Publisher apologizes for the error here.

